# Genistein, a Phytoestrogen in Soybean, Induces the Expression of Acetylcholinesterase via G Protein-Coupled Receptor 30 in PC12 Cells

**DOI:** 10.3389/fnmol.2018.00059

**Published:** 2018-02-27

**Authors:** Etta Y. L. Liu, Miranda L. Xu, Yan Jin, Qiyun Wu, Tina T. X. Dong, Karl W. K. Tsim

**Affiliations:** ^1^Shenzhen Key Laboratory of Edible and Medicinal Bioresources, SRI, The Hong Kong University of Science and Technology, Shenzhen, China; ^2^Division of Life Science, Center for Chinese Medicine, The Hong Kong University of Science and Technology, Hong Kong, Hong Kong

**Keywords:** genistein, neurofilament, AChE, PRiMA, GPR30, 17β-estradiol

## Abstract

Genistein, 4′,5,7-trihydroxyisoflavone, is a major isoflavone in soybean, which is known as phytestrogen having known benefit to brain functions. Being a common phytestrogen, the possible role of genistein in the brain protection needs to be further explored. In cultured PC12 cells, application of genistein significantly induced the expression of neurofilaments (NFs), markers for neuronal differentiation. In parallel, the expression of tetrameric form of proline-rich membrane anchor (PRiMA)-linked acetyl-cholinesterase (G4 AChE), a key enzyme to hydrolyze acetylcholine in cholinergic synapses, was induced in a dose-dependent manner: this induction included the associated protein PRiMA. The genistein-induced AChE expression was fully blocked by the pre-treatment of H89 (an inhibitor of protein kinase A, PKA) and G15 (a selective G protein-coupled receptor 30 (GPR30) antagonist), which suggested a direct involvement of a membrane-bound estrogen receptor (ER), named as GPR30 in the cultures. In parallel, the estrogen-induced activation of GPR30 induced AChE expression in a dose-dependent manner. The genistein/estrogen-induced AChE expression was triggered by a cyclic AMP responding element (CRE) located on the *ACHE* gene promoter. The binding of this CRE site by cAMP response element-binding protein (CREB) induced *ACHE* gene transcription. In parallel, increased expression levels of miR132 and miR212 were found when cultured PC12 cells were treated with genistein or G1. Thus, a balance between production and destruction of AChE by the activation of GPR30 was reported here. We have shown for the first time that the activation of GPR30 could be one way for estrogen or flavonoids, possessing estrogenic properties, to enhance cholinergic functions in the brain, which could be a good candidate for possible treatment of neurodegenerative diseases.

## Introduction

Flavonoids are belonging to a family of polyphenolic compounds, which are also considered as phytestrogen (Miksicek, 1993; Zhu et al., 2007). Flavonoids are commonly found in our daily diet, in particular the major source of which is from fruit and vegetable. Flavonoids possess beneficial effects in a number of disease states, including neurodegenerative disorders, cancer and cardiovascular disease. The brain beneficial effects of flavonoids have been proposed in neuroprotection against neurotoxin stress, promotion of memory, learning and cognitive functions (Zhu et al., 2007, 2009; Choi et al., 2010). Flavonoids could be one of the resources in developing new drugs or food supplements for the prevention of neurodegenerative diseases, e.g., Alzheimer’s disease (AD) and depression. Moreover, the low toxicity of flavonoids in human has been known (Miksicek, 1993).

The modulation of cholinergic activities in neuronal cells could have a potential in finding new therapeutic drugs for neurodegenerative diseases; since cholinergic system is involved in regulating neuronal functions, e.g., memory, conscious, cognition, attention, depression and anxiety (Dagytė et al., 2011). Acetylcholinesterase (AChE) is a polymorphic enzyme that is commonly known for its cholinergic roles by hydrolyzing acetylcholine to choline and acetate in terminating cholinergic signaling at the brain. By alternating splicing at the 3′ end, a single *ACHE* gene generates different isoforms: AChE_R_, AChE_H_, and AChE_T_. Among them AChE_T_ variant is the subunit predominantly expressed in the brain and muscle (Bon and Massoulié, 1997). The localization and oligomerization of AChE_T_ in the brain depends on interaction of its C-terminal peptide (also called tail peptide, t-peptide), with an anchoring protein, proline-rich membrane anchor (PRiMA). The PRiMA-linked AChE produces tetrameric globular form (G4) of the enzyme, which is the predominant and functional form in the brain (Xie et al., 2010; Chen et al., 2011).

Brain beneficial effects of sex hormone estrogen has been widely reported (Coker et al., 2010). An important site of action for estrogen in the brain is targeting at cholinergic system (Newhouse et al., 2013). The effects of estrogen are mediated by two classes of receptors, nuclear estrogen receptors (ERs), e.g., ER α (ERα) and ER β (ERβ), and membrane-bound ERs, e.g., GPR30, ER-X, and Gq-mER. ERα and ERβ are classical nuclear receptors, which could translocate into nucleus and bind to DNA in regulating the expressions of different genes. GPR30 is a seven-transmembrane G-protein coupled receptor, also known as G protein-coupled ER (GPER), which activates the adenylyl cyclase/cAMP-dependent protein kinase A (PKA) signaling pathway (Filardo and Thomas, 2005; Revankar et al., 2005; Thomas et al., 2005). The majority of cholinergic neurons contain GPR30 (Hammond et al., 2011), and therefore which supports the notion that estrogen acting on the brain is mediated by this membrane receptor. Genistein, a common isoflavonoid and a phytestrogen from soybean, is considered as a highly effective agonist for GPR30 (Thomas and Dong, 2006). Here, we aimed to determine the possible role of GPR30, activated by genistein, in regulating the cholinergic enzyme AChE in cultured PC12 cells, a pheochromocytoma derived from rat adrenal medulla.

The regulation of cholinergic enzyme AChE underlies two aspects: production and destruction of AChE transcript and its protein product. MicroRNAs (miRNAs) are small non-coding RNA molecules, with essential functions in RNA silencing and post-transcriptional regulation of gene expression (Krek et al., 2005). The genes of miR132 and miR212 are arrayed in tandem on chromosome and well-studied due to their involvement in brain functions, which are considered as one of the targets of cAMP signaling (Impey et al., 2004; Vo et al., 2005; Shaked et al., 2009). Here, we aimed to reveal the regulation of AChE expression in cultured PC12 cell triggering by genistein-activated G protein-coupled receptor 30 (GPR30). Transcriptional factor and miRNA acting on AChE regulation were elucidated.

## Materials and Methods

### Chemicals

Genistein was purchased from Wako Junyaku (Osaka, Japan). The purity of the chemical was over 98%. For treatments, proper amount of genistein was dissolved in dimethyl sulfoxide (DMSO) forming 100 mM stock solutions and stored at −20°C. G1 and G15 were purchased from Cayman Chemical (Ann Arbor, MI, USA). Reagents, not mentioned, were from Sigma-Aldrich (St. Louis, MO, USA).

### Cell Culture

Rat pheochromocytoma PC12 cell line was obtained from American Type Culture Collection (ATCC, Manassas, VA, USA). The cells were maintained in Dulbecco’s modified Eagle’s medium (DMEM) supplemented with 100 U/mL penicillin, 100 μg/mL streptomycin, 6% fetal bovine serum, and 6% horse serum at 37°C in a humidified 7.5% CO_2_ incubator. Fresh medium was supplied every other day. Reagents for cell cultures were purchased from Invitrogen Technologies (Carlsbad, CA, USA).

### Experimental Design

PC12 cell line was employed in this study; since which was accepted as a common model for studying of neuronal properties. Nerve growth factor (NGF) was served as a positive control for neuronal differentiation and AChE induction. In the study involvement of cAMP pathway, the phosphorylation of cAMP response element-binding protein (CREB) was measured, and H89 (an inhibitor of PKA) was used to block this signaling. To activate GPR30, genistein, G1 (agonist) and G15 (antagonist) were applied onto cultured PC12 cells. The downstream signaling pathway of GPR30 was probed.

### Cell Viability Assay

The cell viability was measured by the colorimetric 3-(4,5-dimethylthioazol-2-yl)-2,5-diphenyltetrazolium bromide (MTT) assay. In brief, cells were cultured in 96-well plate and treated with series concentrations of genistein. After drug treatments for 48 h, MTT solution (0.5 mg/ml) was added into the cultures and then extracted by DMSO solvent. The absorbance was measured at 570 nm. The cell viability was calculated as the percentage of the absorbance value of negative control (without drug treatment), where the absorbance value was set as 100%.

### Ellman Assay

AChE enzymatic activity was determined according to the method of Ellman et al. (1961) with the modification of adding 0.1 mM tetraiso-propylpyrophosphoramide (Sigma) for 5 min, an inhibitor of butyrylcholinesterase activity, to each reaction. Samples were then added to the reaction mixture containing 0.625 mM acetylthiocholine iodide (Sigma) and 0.5 mM 5,5-dithiobis-2-nitrobenzoic acid (Sigma) in 80 mM Na_2_HPO_4_, pH 7.4. For determining the catalytic reaction, the increase in absorbance at 412 nm was recorded, and the specific enzyme activity was expressed as absorbance units/min/g of protein. Protein concentrations were measured throughout by the method of Bradford (1976).

### Sucrose Density Gradient Analyses

Separation of various molecular forms of AChE was performed by sucrose density gradient analysis, as described previously (Noureddine et al., 2007; Xie et al., 2010). In brief, samples of cell extracts (0.2 ml) containing equal amounts of protein (200 μg) were mixed with ALP (6.1 S) and β-galactosidase (16 S), as internal sedimentation markers with known sedimentation coefficient, layered onto continuous sucrose gradients (5%–20%) in a detergent-containing buffer (10 mM HEPES, pH 7.5, 1 mM EDTA, 1 mM EGTA, 0.2% Triton X-100, and 1 M NaCl), which were prepared in 12-ml polyallomer tubes. Centrifugation was performed at 38,000 rpm in a SW41 Ti Rotor (Beckman) at 4°C for 16 h. Approximately 45 fractions were collected from the bottom of the tubes and assayed for AChE, ALP, and β-galactosidase activities, as described previously (Xie et al., 2010; Chen et al., 2011), allowing the determination of the sedimentation coefficients of the different AChE containing fractions and of their relative activities. The sedimentation coefficients of G1 and G4 isoforms of AChE were approximately 4.3 S and 10.2 S, respectively.

### Neurite Outgrowth Assay

The images of cultured PC12 cells were captured by a light microscope (Zeiss Group, Jena, Germany) equipped with a phase-contrast condenser, 10× objective lens and a digital camera (Zeiss Group) with the manual setting. Approximately 100 cells were counted from at least 10 randomly chosen visual fields for each culture. The cells were analyzed for number and length of neurite by using the SPOT basic software (Diagnostic Instruments, Sterling Heights, MI, USA). If one or more neurites was longer than diameter of cell body, the cell was considered as differentiated.

### Real-Time Quantitative PCR

Total RNA was extracted by RNAzol^®^ RT Reagent (Molecular Research Center, Cincinnati, OH, USA) according to the manufacturer’s protocol. Isolated RNAs were reverse transcribed into cDNAs according to the manufacturer’s instructions (Invitrogen). Real-time quantitative PCR for the target genes was performed on equal amounts of cDNA by using Roche SYBR Green Master mix with Rox reference dye, according to the manufacturer’s instructions (Winer et al., 1999). Each sample was run in triplicate. Transcript expression level of genes was calculated using 18S rRNA as the internal control. Primers employed in RT-PCR were as follows: AChE catalytic subunit (5′-AAT CGA GTT CAT CTT TGG GCT CCC CC-3′ and 5′-CCA GTG CAC CAT GTA GGA GCT CCA-3′; NM_015831); PRiMA (5′-TCT GAC TGT CCT GGT CAT CAT TTG CTA C-3′ and 5′-TCA CAC CAC CGC AGC GTT CAC-3′; NM_178023); 18S rRNA (5′-GAC TGT TAT GGT CAA GGT GAA-3′ and 5′-GAT AGT CAA GTT CGA CCG TC-3′; NR_003286). For detection of miRNAs, miRNAs from cultured PC12 cells were isolated by miRcute miR Isolation Kit (Tiangen Biotech, Beijing, China) according to the manufacturer’s instruction. In brief, cells were lysed with Buffer MZ/chloroform reagent (the similar function as Trizol/chloroform reagent). The mixture was centrifuged at 12,000 × *g* for 15 min at 4°C, and the upper aqueous phase was transferred to an absorption column in the miRNA extraction kit. The obtained mixture was then processed according to the instruction. The miRNAs were reverse transcribed to cDNA using the miRcute Plus miRNA First-Strand cDNA Synthesis Kit (Tiangen Biotech) according to the manufacturer’s instruction. The qPCR analysis was performed on equal amounts of cDNA by using miRcute Plus miRNA qPCR Detection Kit (Tiangen Biotech). Each sample was run in triplicate. Expression levels of the miRNAs were calculated using small nuclear RNA U6 (snRNA) as an internal reference for normalization. Forward and reverse primers were provided by Tiangen Biotech with product code CD201-T (rno-miR-U6, rno-miR-132, rno-miR-212). The comparative ΔΔCt method was used to determine levels of each mRNA (normalized to 18S) and miRNA (normalized to RNU6B).

### SDS-PAGE and Western Blot Analyses

PC12 cell cultures were collected in the low salt lysis buffer (10 mM HEPES, pH 7.5, 100 μM NaCl, 1 mM EDTA, 1 mM EGTA, 0.5% Triton X-100, 5 mM benzamidine HCl, 10 μM aprotinin, and 10 μM leupeptin), followed by centrifugation at 13.2 × 104 rpm for 15 min at 4°C. Total protein content was measured by using Bradford’s method. Samples are homogenized to equal amounts of total protein. The homogenates were treated with 2× direct lysis buffer and boiled for 10 min before performing on the 8% gel electrophoresis (Chen et al., 2011). The gel was run in 1× SDS-PAGE running buffer (25 mM Tris, pH 8.3, 0.192 M glycine, 0.1% SDS) at 60V. After the electrophoresis separation, the proteins in SDS-PAGE were transferred to a nitrocellulose membrane, using a Mini Trans-Blot^®^ Cell at 40 V, 0.1 A for 16 h in 1× transfer buffer (24 mM Tris, 0.192 M glycine, 15% ethanol). The membrane was stained with Ponceau S to confirm the transfer and equal loading of the samples. Membrane was blocked with 5% dry milk in TBS-T for 1 h at room temperature then incubated in primary anti-AChE antibodies (1:500, Santa Cruz Biotechnology, Santa Cruz, CA, USA) for overnight at 4°C. After intensive washing with TBS-T, HRP-conjugated anti-goat secondary antibody (Invitrogen) with 1:5000 dilutions were added and incubated for 2 h at room temperature. The immune complexes were visualized using ECL method. The expression level of proteins was calculated using glyceraldehyde 3-phosphate dehydrogenase (GAPDH) as an internal control.

### DNA Constructions and Transfection

The DNAs (~2.2 kb) encompassing human AChE promoter with and without the cyclic AMP responding element (CRE)-binding site, human PRiMA promoter was sub-cloned into pGL3 vector (BD Biosciences Clontech, Palo Alto, CA, USA) upstream of a luciferase gene, designated as pAChE-Luc, pAChE_ΔCRE_-Luc, and pPRiMA-Luc. The DNA construct of CRE sequences tagged with a luciferase gene (pCRE-Luc) was from BD Biosciences Clontech (Palo Alto, CA, USA). Transient transfection of PC12 cells with the cDNA constructs was performed with jetPRIME^®^ reagent, according to the manufacturer’s instruction. The transfection efficiency was consistently 30%–40% in PC12 cell culture, as determined by another control plasmid having a β-galactosidase gene under a cytomegalovirus (CMV) enhancer promoter. Luciferase assay was performed by using a commercial kit (Tropix Inc., Bedford, MA, USA). In brief, cell cultures were washed with PBS and resuspended in 100 mM potassium phosphate buffer (pH 7.8) containing 0.2% Triton X-100 and 1 mM dithiothreitol. Thirty microliter of lysate per sample was used in luciferase assay. The luminescent reaction was quantified in a Tropix TR717 microplate luminometer, and the activity was expressed as absorbance (up to 560 nm) per mg of protein. Protein concentrations were measured routinely by the Bradford method with a kit from Bio-Rad (Hercules, CA, USA).

### Statistical Analyses

Each result represented the mean ± SEM, each with triplicate samples. Comparisons of the means for untreated control cells and treated cells were analyzed using one-way ANOVA and Student’s *t*-test. Significant values were indicated by *p < 0.05; **p < 0.01; and ***p < 0.001.

## Results

### Genistein Promotes Expression of Neurofilaments in Cultured PC12 Cells

The effect of genistein on the cell viability in PC12 cells was first determined by MTT assay. By compared to the negative control (DMSO treatment), genistein treatment at concentrations not more than 10 μM showed no significant effect on cell viability (Supplementary Figure S1), indicating that the current treatments were not cytotoxic to neuronal cells. Hence, the concentrations not more than 10 μM were chosen in the following assays.

**Figure 1:**
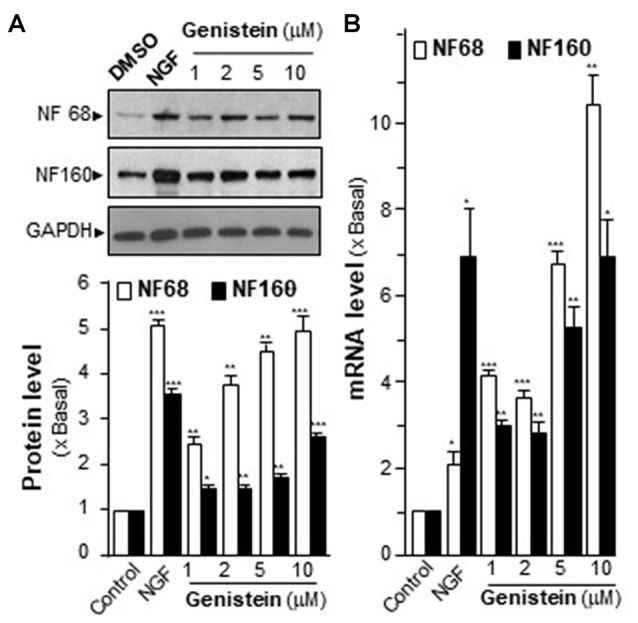
Effect of genistein on neurofilaments (NFs) on cultured PC12 cells. **(A)** Cultured PC12 cells were treated with genistein for 48 h, and nerve growth factor (NGF; 20 ng/ml) was a positive control. The protein of cultures was collected to determine the expression levels of NFs (NF68 and 160) by western blotting (upper panel). The expression level was calculated using glyceraldehyde 3-phosphate dehydrogenase (GAPDH) as an internal control. Quantification plot was shown in histograms (lower panel). **(B)** The total RNA of PC12 cells was collected to determine the mRNA expression levels of NFs (NF68 and NF160). Transcript expression level of genes was calculated using 18S rRNA as an internal control. Data are expressed as the fold of basal value where control value (control culture treated with 0.02% dimethyl sulfoxide, DMSO) is set as 1, mean ± SEM, *n* = 5, each with triplicate samples. *p < 0.05; **p < 0.01; ***p < 0.001.

Neurofilaments (NFs) are major compositions of neurite. NF68 (at ~68 kDa) and NF160 (at ~160 kDa) are two major neurofilament subunits, which are named based upon their apparent molecular masses on SDS-PAGE gel. NF68 is expressed at the beginning of neurite outgrowth, and NF160 is expressed shortly after the emergence of neurite formation. As shown in Figure 1A, the genistein-treated PC12 cells showed significant induction of NFs in a dose-dependent manner. At the maximal induction, the levels of NF68 and NF160 were increased by ~5-fold and ~3-fold, respectively. The mRNA levels of NFs were also measured. In a dose-dependent manner, genistein significantly increased the transcripts encoding NF68 and NF160 to ~10-fold and ~6-fold, respectively (Figure 1B). In general, the increase of neurofilament expression was associated with neurite outgrowth. However, the genistein-treated PC12 cells did not show increase in neurite length (Supplementary Figure S2). NGF served as a control in inducing neurofilament and neurite out-growth. Nevertheless, genistein was able to trigger part of signaling cascades during the neuron differentiation.

### Genistein Enhances the Expression of AChE in PC12 Cells

**Figure 2:**
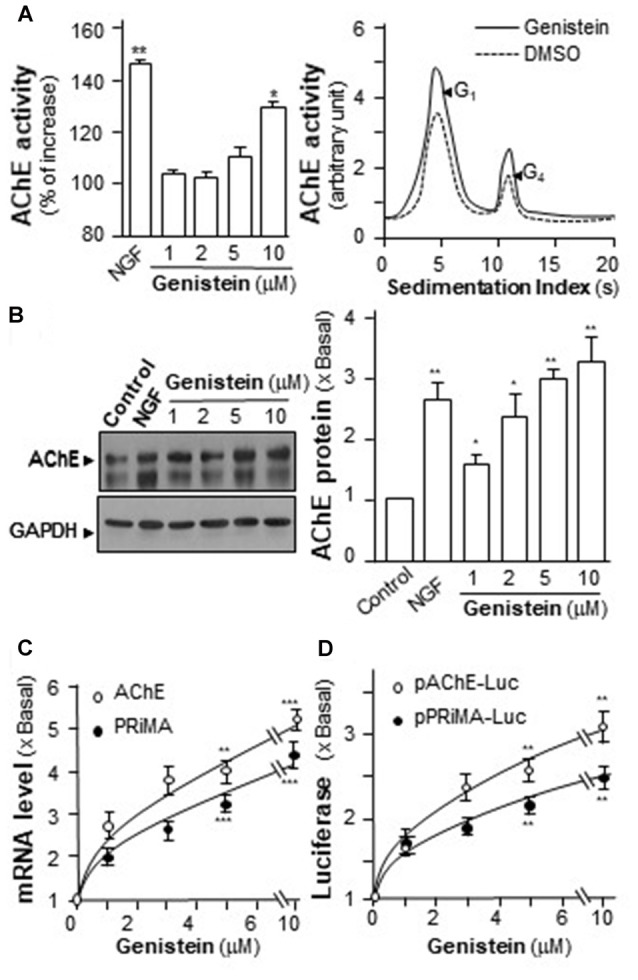
Genistein induces proline-rich membrane anchor (PRiMA)-linked acetylcholinesterase (AChE) in cultured PC12 cells. **(A)** Cultured PC12 cells were treated with genistein for 48 h, and NGF (20 ng/ml) served as a positive control. Cell lysates were collected for Ellman assay (left panel) and sucrose density gradient analysis (right panel). **(B)** Protein lysates of the cultures as in **(A)** were performed Western blot analysis (left panel). The expression level of proteins was calculated. Quantification plot was shown in histograms (right panel). **(C)** Total RNAs was extracted from cultures to perform quantitative PCR. Values were normalized with 18S rRNA.** (D)** Cultured PC12 cells were transiently transfected with two promoter constructs, pAChE-Luc and pPRiMA-Luc for 24 h. Cell lysates were collected for luciferase assay. Values are expressed as the fold, or percentage, of increase to basal reading, and are in means ± SEM, *n* = 5, each with triplicate samples. *p < 0.05; **p < 0.01; ***p < 0.001.

The increase of AChE activity has been associated with the differentiation of neuron, i.e., the NGF-induced AChE expression (Figure 2A). Similarly, genistein application increased AChE activity in a dose-dependent manner in cultured PC12 cells (Figure 2A). Genistein, more than 5 μM, induced the AChE activity to a maximal induction of ~35% at 10 μM. By sucrose density gradient analyses, the amounts of G1 and G4 isoforms of AChE were induced in genistein-treated cultures (Figure 2A). The result suggested that genistein could increase the assembly of PRiMA-linked G4 AChE in PC12 cells. The protein level of AChE catalytic subunit was determined by western blotting. The expression of AChE catalytic subunit, two bands at ~60 kDa and ~68 kDa, was markedly induced by over 3-fold at 10 μM of genistein, and the induction was in a dose-dependent manner (Figure 2B). NGF, a positive control here, induced both AChE activity and protein in PC12 cells.

To determine the regulatory role of genistein on *AChE* and *PRiMA* gene transcriptions, the promoters tagged with a luciferase gene, i.e., pAChE-Luc and pPRiMA-Luc, were employed here. The promoter constructs were transfected into cultured PC12 cells, and then which were treated with genistein for 2 days. The transcripts encoding AChE and PRiMA were significantly up regulated by genistein application in a dose-dependent manner: the transcript induction at 10 μM of genistein was at ~5 folds (Figure 2C). The transcript encoding PRiMA was also up regulated by ~4-fold at the maximum. In parallel, the luciferase activities, driven by the promoters (pAChE-Luc and pPRiMA-Luc), were increased by the challenge of genistein (Figure 2D).

### Genistein-Induced AChE Is Blocked by PKA Inhibitor

**Figure 3:**
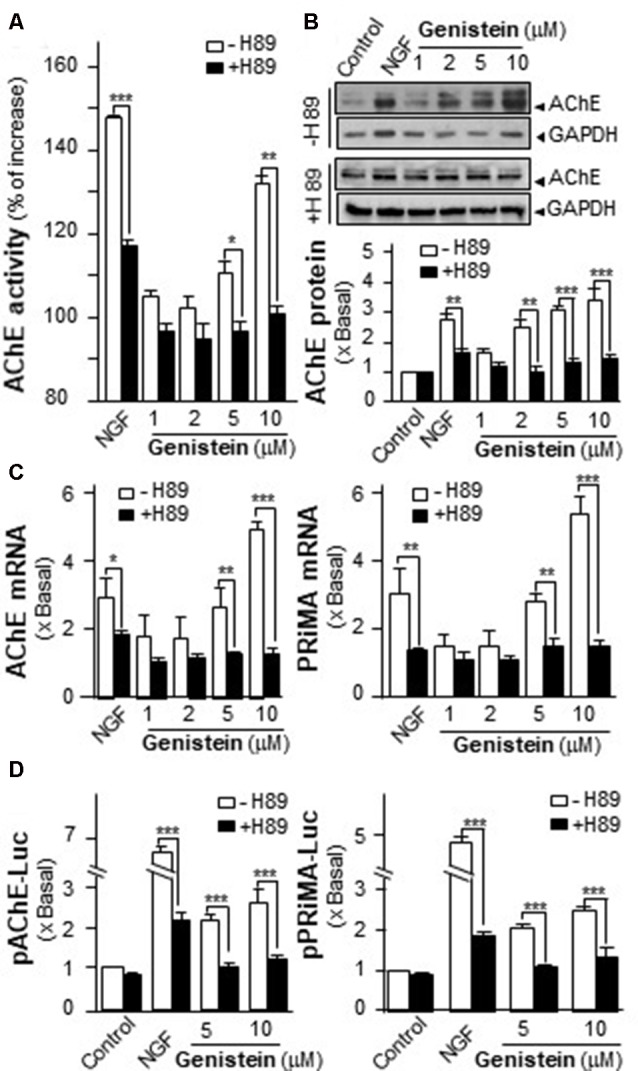
Protein kinase A (PKA) inhibitor blocks the genistein-induced AChE and PRiMA expression. **(A)** Cultured PC12 cells were pre-treated with PKA inhibitor H89 (5 μM) for 3 h. Then, treated with genistein for 48 h. Cell lysates were collected for Ellman assay. Data are expressed as the percentage of increase where control value was set as 0. **(B)** Protein lysates of the cultures in **(A)** were performed Western blot analysis (upper panel). Quantification plot was shown in histograms (lower panel). **(C)** Treatment of the cultures as in **(A)**. Total RNAs was extracted from cultures to determine the mRNA levels of AChE (left panel) and PRiMA (right panel). Values were normalized with 18S rRNA. **(D)** Cultured PC12 cells were transiently transfected with two promoter constructs, pAChE-Luc and pPRiMA-Luc, for 24 h and treated with genistein and H89. Cell lysates were collected for luciferase assay. Data are expressed as the fold of basal value where control value is set as 1, mean ± SEM, *n* = 5, each with triplicate samples. *p < 0.05; **p < 0.01; ***p < 0.001.

An increase in intracellular level of cAMP has been shown to induce neuronal differentiation (Sánchez et al., 2004) and to regulate PRiMA-linked G4 AChE in terms of gene transcription and molecular assembly in cultured PC12 cells (Choi et al., 2008). To probe the possible role of cAMP in genistein-induced AChE expression, cultured PC12 cells were pre-treated with PKA inhibitor H89 before the treatment with genistein. The pre-treatment of H89 blocked fully the genistein-induced AChE activity and protein (Figures 3A,B). In parallel, H89 further blocked fully the genistein-induced expression of transcripts encoding AChE catalytic subunit and PRiMA (Figure 3C). This blockage was also revealed by the genistein-induced promoter activities, i.e., pAChE-Luc and pPRiMA-Luc (Figure 3D). The full blockage by PKA inhibitor suggested the signaling, triggered by genistein, should be mediated by a cAMP-dependent cascade. Serving as a control, the inhibitor of PKA showed partial blockage to NGF-induced AChE expression.

### Genistein Induces the Phosphorylation of CREB in Cultured PC12 Cells

**Figure 4:**
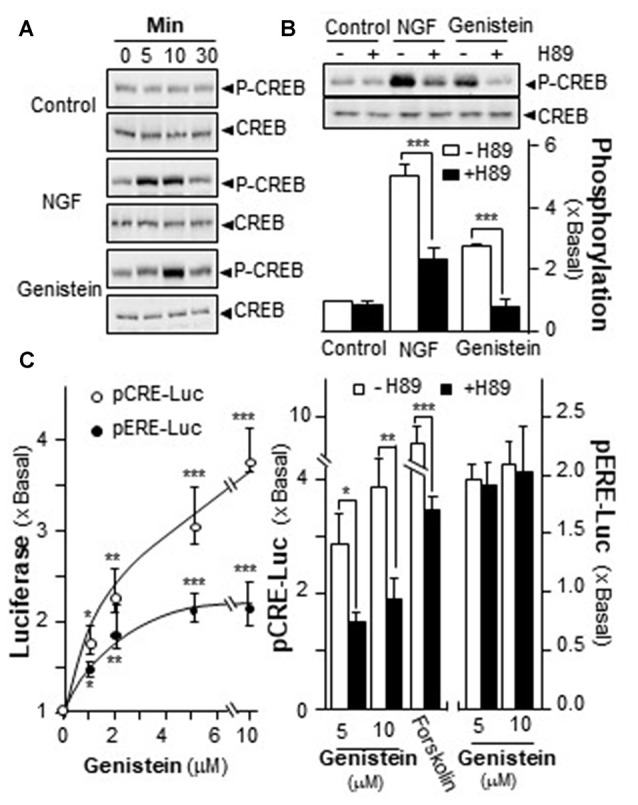
Genistein induces the phosphorylation of cAMP response element-binding protein (CREB). **(A)** Cultured PC12 cells, serum starved for 5 h, were treated with NGF at 20 ng/mL and genistein at 10 μM for different time. Total CREB and phosphorylated CREB (~42 kDa) were revealed by using specific antibodies. **(B)** Cultured PC12 cells, serum starved for over 5 h, were pre-treated with or without PKA inhibitor, H89 (5 μM) for 3 h prior to the treatment with NGF or genistein for 10 min as in **(A)**. Total CREB and phosphorylated CREB (~42 kDa) were revealed by using specific antibodies (upper panel). Quantification plot was shown in histograms (lower panel). **(C)** Cultured PC12 cells were transiently transfected with two promoter constructs, pCRE-Luc and pERE-Luc, for 24 h and pre-treated with or without H89. Then, genistein was treated for 2 days. Cell lysates were collected for luciferase assay (left panel). Cultured PC12 cells were transiently transfected with the constructs for 24 h and pre-treated with or without H89 (right panel). Cell lysates were collected for luciferase assay. Data are expressed as the fold of basal value where control value is set as 1, mean ± SEM, *n* = 5, each with triplicate samples. *p < 0.05; **p < 0.01; ***p < 0.001.

Increase of intracellular level of cAMP has been shown to regulate PRiMA-linked G4 AChE in cultured PC12 cells. Activation of cAMP signal leads to an increase of PKA, which travels into nucleus and phosphorylates CREB (Gonzalez and Montminy, 1989). In order to find out the potency of genistein in stimulating AChE expression via the cAMP pathway, the phosphorylation of CREB at ~43 kDa was measured. NGF induced the phosphorylation of CREB robustly, serving as a positive control. Genistein was applied onto the serum-starved PC12 cultures, which induced CREB phosphorylation: the induction was transient having a maximum at 10 min (Figure 4A). The total amount of CREB remained unchanged. The CREB phosphorylation, induced by NGF or genistein, could be blocked by H89 (Figure 4B). The blockage in case of genistein was more robust than that of NGF.

A luciferase-reporter construct (pCRE-Luc), containing three copies of CRE, derived from the promoter, and tagged upstream of a luciferase gene was applied to investigate the transcriptional activation of CRE. Forskolin, an adenylate cyclase activator has been shown to stimulate adenylate cyclase in PC12 cells, as such to raise intracellular cAMP level. Forskolin was used as a positive control here to activate pCRE-Luc. Genistein is a well-known activator for ER. Thus, a DNA construct having estrogen responsive element tagged upstream of luciferase (pERE-Luc) was applied to investigate the estrogenic properties of genistein. In pCRE-Luc or pERE-Luc transfected PC12 cells, genistein induced the luciferase activity in a dose-dependent manner, maximal inductions were at ~250% and ~100%, respectively (Figure 4C, left panel). The induction of pCRE-Luc was much higher than that of pERE-Luc. To further confirm the possible mechanism of genistein in cAMP-dependent pathway, the pCRE-Luc and pERE-Luc transfected PC12 cells were pre-treated with H89. After the treatment, the genistein-induced transcriptional activity of pCRE-Luc was markedly decreased. In contrast, the transcriptional activity of pERE-Luc was not changed by the inhibitor (Figure 4C). This discrepancy of H89 sensitivity suggested the distinction of two signaling pathways that could be triggered by genistein, i.e., cAMP-dependent signaling via GRP30 and estrogen responsive element via nucleus ER.

### Activation of GPR30 Induces the Expression of AChE

**Figure 5:**
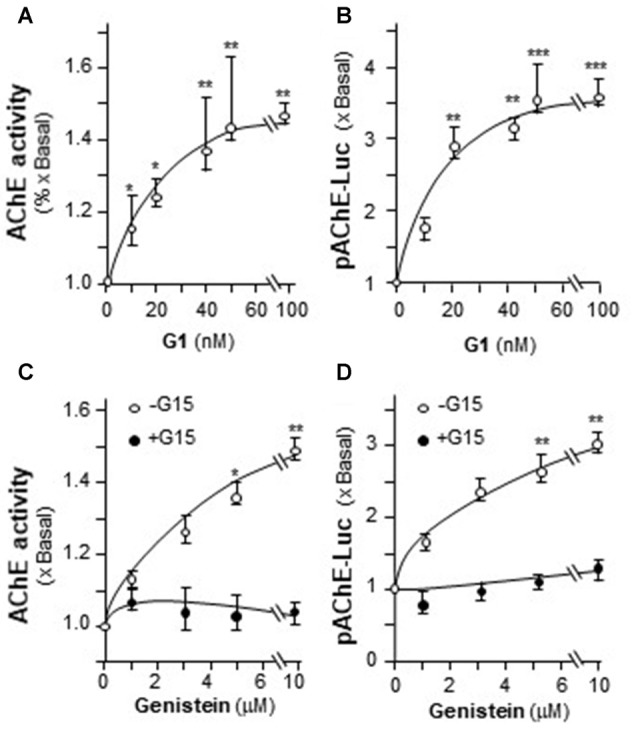
The effect of G protein-coupled receptor 30 (GPR30) agonist on AChE expression. **(A)** Cultured PC12 cells were treated with GPR30 agonist G1 for 48 h. Cell lysates were collected for Ellman assay. **(B)** Cultured PC12 cells were transiently transfected with pAChE-Luc for 24 h and treated with G1. Cell lysates were collected for luciferase assay. **(C)** Cultured PC12 cells were pre-treated with or without GPR30 antagonist G15 (10 μM) for 3 h. Then, treated with genistein for 48 h. Cell lysates were collected for Ellman assay. **(D)** Cultured PC12 cells were transiently transfected with pAChE-Luc for 24 h and treated as in **(C)**. Cell lysates were collected for luciferase assay. Data are expressed as the fold of basal value where control value is set as 1, mean ± SEM, *n* = 5, each with triplicate samples. *p < 0.05; **p < 0.01; ***p < 0.001.

The possible involvement of ER in genistein-induced AChE in PC12 cells was determined. Estrogen activates GPR30 and triggers PKA pathway (Filardo and Thomas, 2005; Thomas et al., 2005). Here, the GPR30 agonist (G1) and antagonist (G15) were used. Cultured PC12 cells were treated with different concentrations of G1. As shown in Figure 5A, G1 application increased AChE activity in a dose-dependent manner in cultured PC12 cells, and maximal induction was at ~50%. In parallel, the luciferase activity of AChE (pAChE-Luc) was increased by the challenge of G1 (Figure 5B). At 100 nM of G1, the increased activity of pAChE-Luc was up to ~3.5-fold, compared to control. The signaling of genistein-induced AChE expression was probed here. In the cultures, the pre-treatment of G15 blocked fully the genistein-induced AChE activity (Figure 5C), as well as the activity of pAChE-Luc (Figure 5D). These results suggested the direct role of GPR30 in genistein-mediated AChE regulation.

### Estrogen Activates the Transcription of PRiMA-Linked AChE via GPR30

**Figure 6:**
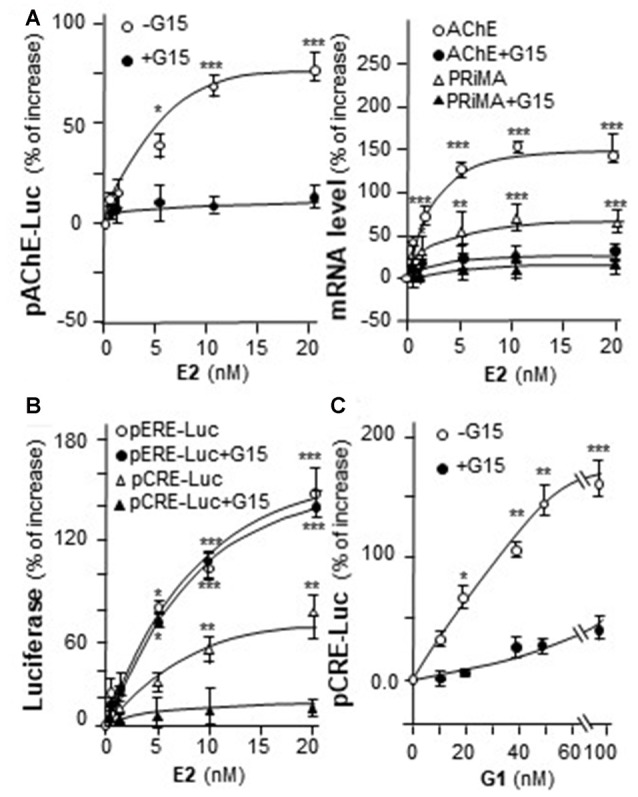
E2 induces the transcription of AChE. **(A)** Cultured PC12 cells were transiently transfected with pAChE-Luc for 24 h and pre-treated with or without G15 (10 μM). Then, treated with E2 for 48 h. Cell lysates were collected for luciferase assay (left panel). Total RNAs was extracted from cultures to determine the mRNA levels of AChE and PRiMA (right panel). Values were normalized with 18S rRNA. **(B)** Cultured PC12 cells were transiently transfected with pCRE-Luc or pERE-Luc for 24 h and pre-treated with or without G15 (10 μM). Then, E2 was treated for 2 days. Cell lysates were collected for luciferase assay.** (C)** Cultured PC12 cells were transiently transfected with pCRE-Luc for 24 h and pre-treated with or without G15 (10 μM). Then, treated with G1 for 48 h. Cell lysates were collected for luciferase assay. Data are expressed as the percentage of increase where control value is set as 0. mean ± SEM, *n* = 5, each with triplicate samples. *p < 0.05; **p < 0.01; ***p < 0.001.

Genistein is not an endogenous ligand for GRP30 receptor. Thus, the endogenous ligand E2 was being tested here. E2 application increased pAChE-Luc activity in a dose-dependent manner in cultured PC12 cells. The pre-treatment of G15 blocked fully this induction (Figure 6A, left panel). In parallel, the transcripts encoding AChE and PRiMA were significantly up regulated by E2 application in dose-dependent manners, which were fully blocked by G15 pre-treatment (Figure 6A, right panel). Cultured PC12 cells were transient transfected with pCRE-Luc or pERE-Luc, then the cells were challenged with E2 or G1. E2 induced pERE-Luc activity much higher than pCRE-Luc. The pre-treatment of G15 blocked E2-induced pCRE-Luc activity rather than pERE-Luc (Figure 6B). Indeed, E2 is a much stronger agonist for intracellular ER than that in GRP30. In addition, G1 increased pCRE-Luc activity in a dose-dependent manner in cultured PC12 cells. This induction was blocked by pre-treatment of G15 (Figure 6C).

### Genistein Induces AChE via CRE Element Binding Site

**Figure 7:**
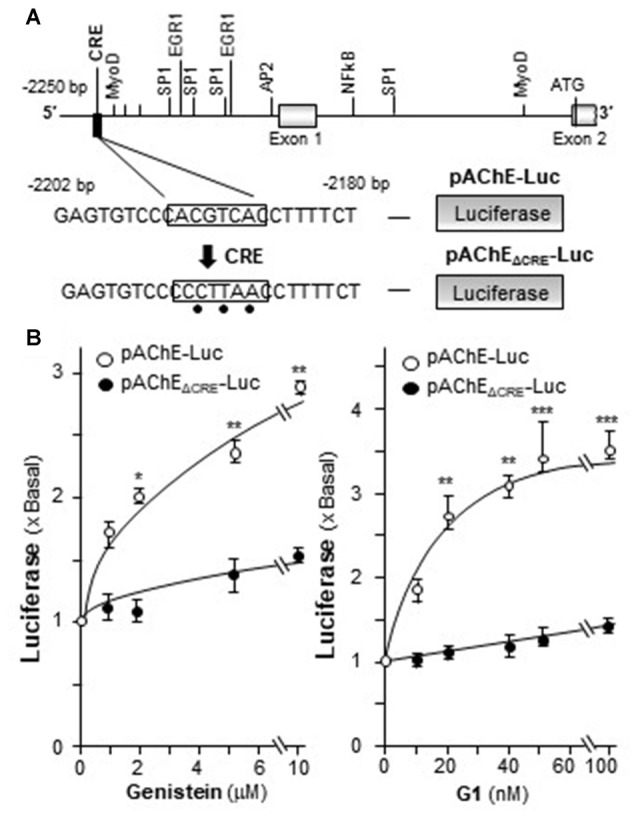
Genistein regulates AChE transcription via cyclic AMP responding element (CRE) binding site. **(A)** Deletion of the CRE-binding site on human *ACHE* promoter was shown. A schematic diagram of human *ACHE* promoter with key transcription elements was shown. The CRE-binding site sequence (CAC GTC A) was mutated into CCC TTA A on the promoter. **(B)** Cultured PC12 cells were transiently transfected with pAChE-Luc and its mutant pAChE_ΔCRE_-Luc for 24 h before the application of genistein (left panel) or G1 (right panel). The cultures were collected for luciferase assay. Data are expressed as the fold of basal value where control value is set as 1. mean ± SEM, *n* = 5, each with triplicate samples. *p < 0.05; **p < 0.01; ***p < 0.001.

In mammalian *ACHE* gene, the binding site for CRE element was revealed and demonstrated to play an important role in cAMP-mediated AChE expression in neuron (Wan et al., 2000; Choi et al., 2008; Xu et al., 2017). Human AChE promoter has only one CRE binding site at ~2 kb upstream of the ATG start site (Wan et al., 2000). To confirm the induction effect of CRE on the *ACHE* gene, pAChE_ΔCRE_-Luc, having mutation on the CRE-binding site of the AChE promoter, was used in transfected PC12 cells. The CRE-binding site sequence (CAC GTC A) was mutated into CCC TTA A on the human promoter (Figure 7A). The application of genistein in the transfected cells did not show any induction on pAChE_ΔCRE_-Luc activity (Figure 7B). In parallel, the induction, triggered by G1, was fully blocked by the CRE mutation on the promoter.

### Activation of GPR30 Induces the Expression of miR132 and miR212

**Figure 8:**
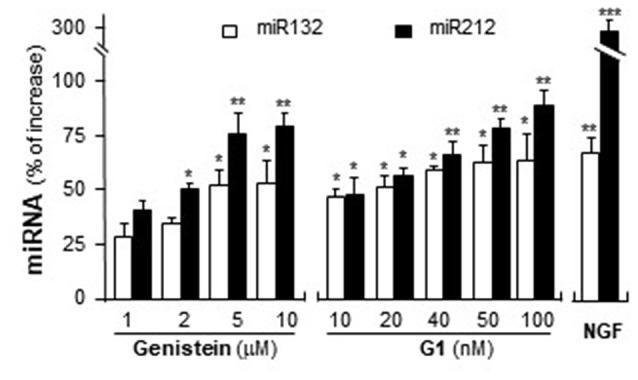
Genistein and G1 induce the expression of miR132 and miR212. Cultured PC12 cells were treated with genistein, G1, or NGF (20 ng/ml) for 48 h. MicroRNAs (MiRNAs) were collected from cultures to determine the levels of miR132 and miR212. Values were normalized with RNU6B. Data are expressed as the percentage of increase, where control value is set as 0. mean ± SEM, *n* = 3, each with triplicate samples. *p < 0.05; **p < 0.01; ***p < 0.001.

MiR132 and miR212 are considered as the CREB-regulated miRNAs (Impey et al., 2004; Vo et al., 2005). Activation of GPR30 triggers PKA pathway (Filardo and Thomas, 2005; Thomas et al., 2005) and then phosphorylates CREB (Gonzalez and Montminy, 1989). Hence, the potential effects of activation of GPR30 on the expression of miR132 and miR212 were determined here. Cultured PC12 cells were treated with genistein and G1. As shown in Figure 8, genistein application increased the expression of miR132 and miR212, and maximal inductions were at ~50% and ~75%, respectively. In parallel, the expressions of miR132 and miR212 were induced by the challenge of G1. At 100 nM of G1, the expressions increased ~60% and ~90%, respectively. NGF at 20 ng/ml, as a control, induced miR132 at ~70%. A robust induction was found on the effect of NGF on miR212, i.e., ~3-fold change as compared to control.

## Discussion

NGF was the most efficacious neurotrophic factor to induce neuronal differentiation and to prevent atrophy of cholinergic neurons in patients with neurodegenerative diseases (Lin et al., 2010). However, this polypeptide cannot easily cross the blood brain barrier (BBB) and is metabolized by peptidases when administered peripherally (Hur et al., 2009). There is mounting evidences to support the use of non-peptidic factors against neuro-degenerative disorders, and phytochemical is a popular choice. Amongst these natural chemicals, phytestrogen is known to induce neuronal differentiation and regulate cholinergic activities in the brain (Omoni and Aluko, 2005). Genistein is a phytestrogen mainly found in lupin, soybean, kudzu and psoralea, which has been shown to have direct binding to ERs (Dixon and Ferreira, 2002). Genistein has a wide range of potential health effects, e.g., anti-oxidant, anti-cancer, relieving symptoms associated with post-menopausal conditions, and preventing cardiovascular disease (Andersen et al., 2003). This phytestrogen has been demonstrated to be BBB permeable having promising benefits on the brain, and thus which could be a good candidate for treatment of neurodegenerative diseases (File et al., 2001). In the brain, genistein can improve spine thickness, cognitive function, synapse development and regulating the transcription factor of neurotrophic genes in the hippocampal region of adult animals (Bagheri et al., 2011). Our previous work in cultured rat astrocytes showed that genistein induced synthesis and secretion of neurotrophic factors, e.g., NGF, glial-derived neurotrophic factor (GDNF) and brain-derived neurotrophic factor (BDNF): the induction was shown to be triggered by intracellular ER in time- and dose-dependent manners (Xu et al., 2013). In addition, the anti-oxidant property of genistein suggested its effective therapeutic strategy for treatment of AD (Zeng et al., 2004). However, the aforementioned effects of genistein in various tissues need varied concentrations, and only some of these identified concentrations are relevant in people consuming soy-rich diet. Thus, the administration of genistein at higher dose may be needed.

In neuroblastoma cells, application of genistein increased AChE activity and neurite outgrowth: this effect of genistein was proposed to be mediated by a tyrosine protein kinase (Mufson et al., 2008). In line to this observation, the genistein-induced AChE activity was not found in a “genistein receptor” free mouse erythroleukemia cell line (Rocchi et al., 1994). Isoda et al. (2002) reported that application of genistein induced the activity of AChE in cultured PC12 cells. However, the underline mechanism was not revealed. The aforementioned evidence therefore suggested that genistein could be a strong cholinergic enhancer. Indeed, several lines of evidence suggest that AChE playing role in the CNS, happened in case of depression, as well as in the impairment of behavior and arousal (Dagytė et al., 2011). In line to this notion, the loss of AChE activity was observed in mild cognitive impairment and in early AD (Mufson et al., 2008). In view of these brain beneficial functions, genistein could be a good candidate for treatment of neurodegenerative diseases via regulation of AChE.

There are two aspects regarding the regulation of AChE: production and destruction of AChE. The activation of GPR30, triggered by genistein or G1, induced the mRNA and promoter activity of AChE, which indicated the inductive effect on *ACHE* transcription. According to our results, this induction was regulated by the binding of CREB transcriptional factor onto the *ACHE* gene. In addition, miR132 and miR212 were considered as targets of CREB (Impey et al., 2004; Vo et al., 2005). The induction of miR132 and miR212 were identified here by the activation of GPR30. MiR132, or together with miR212, has been considered as translational repression and/or degradation of AChE mRNA (Shaked et al., 2009). Even though the suppressive role of miR132 and miR212 have not been shown here in cultured PC12 cells, the up regulation of which in genistein-treated cells could regulate AChE expression contrary to that of CREB. Thus, a balance of production and destruction of AChE mRNA and protein under the signaling of CPR30 activation should be addressed.

Activation of GPR30 could be one way for estrogen, or flavonoids possessing estrogenic properties, to enhance the cholinergic functions in the brain. Indeed, the majority of cholinergic neurons contain GPR30 (Hammond et al., 2011). Activation of GPR30 by G1 increased potassium-stimulated ACh release in hippocampus, and which enhanced the acquisition of delayed matching-to-position task: this effect was blocked by G15, a selective GPR30 antagonist (Hammond et al., 2011). GPR30 activation, but not ERα and ERβ, rapidly enhanced the social transmission of food preferences (STFP) task, a function triggered by cholinergic neurons (Ervin et al., 2015). In addition, estrogen, mediated by GPR30, was shown to be a neuroprotective agent both *in vivo* and *in vitro* (Gingerich et al., 2010; Lebesgue et al., 2010). Here, we showed the genistein-induced AChE expression in PC12 cells could be fully blocked by pre-treatment of G15, a selective GPR30 antagonist, suggesting a direct involvement of GPR30. In GPR30-mediated AChE expression, the receptor activation, triggered by E2 or G1 or genistein, can rapidly activate the production of cAMP (Filardo and Thomas, 2005), which subsequently gives rise to activation of transcription factor CREB. Then, the transcription factor CREB will bind to the CRE binding site on *ACHE* gene that leads to the transcription of AChE. Have to point out that in our study, genistin, a genistein β-glycoside, showed no effect on the induction of AChE in cultured PC12 cells (Liu et al., unpublished result), suggesting a structural requirement for genistein binding to GPR30.

Activation of GPR30 by genistein in tumor cell is well studied. In breast cancer cells, application of E2 or genistein was able to induce c-fos up regulation through GPR30 (Maggiolini et al., 2004). Similarly, in thyroid cancer cells, E2 and genistein could activate the MAPK pathway via GPR30, as a result to stimulate c-fos expression and growth response (Vivacqua et al., 2006). In periodontal ligament cells, genistein significantly delayed the IL-1β-induced activation of MAPKs via GPR30 (Luo et al., 2012). In line with our study, the aforementioned GPR30 activation was triggered by genistein at μM concentration. Besides genistein, other flavonoids, or phenolic phytochemicals, have been demonstrated to bind GPR30, e.g., oleuropein, hydroxytyrosol, resveratrol, equol, quercetin, tectoridin, daidzein and apigenin (Prossnitz and Arterburn, 2015). In accord to our current study, quercetin showed the induction of AChE in cultured PC12 cells (Liu et al., unpublished result). The identification of these AChE-inducing natural products could be very useful in finding potential drugs or food supplements for possible new therapeutic opportunities in neurodegenerative diseases.

## Author Contributions

EYLL designed and performed most of the experiments, analyzed the results and wrote most of the article. MLX helped to amplify the plasmids used in this article. YJ helped to culture PC12 cells. QW designed and performed the PCR for *NF68, NF160, ACHE* and *PRiMA* genes. TTXD prepared all the chemicals. KWKT conceived the idea for the project and wrote the article with EYLL.

## Conflict of Interest Statement

The authors declare that the research was conducted in the absence of any commercial or financial relationships that could be construed as a potential conflict of interest.

## Supplementary Material

FIGURE S1 Effect of genistein on the viability of PC12 cells. Cultured PC12 cells were treated with the different doses of genistein for 48 h. Cell viability (using the colorimetric MTT assay) was performed. No significant increase in cell viability was observed up to 10 μM. Values are in the fold of change as compared to control, and in mean ± SEM, *n* = 5, each with triplicate samples. ***p* < 0.01.

FIGURE S2 The genistein-treated PC12 cells do not show increase in neurite length. Genistein and nerve growth factor (NGF) were applied onto cultured PC12 cells for 48 h. Cells were fixed with ice-cold 4% paraformaldehyde. The images were examined under microscope, and a digital camera was used to capture the images with manual setting (left panel). The % of differentiated cells was counted as described in “Materials and Methods” section (right panel). Representative images are shown, bar = 50 μm. Dimethyl sulfoxide (DMSO) was served as the negative control. Values were expressed as % of cells in 100 counted cells, Mean ± SEM, *n* = 3. Each with triplicate samples. ****p* < 0.001 as compared to the control group.

## Abbreviations

AChE: acetylcholinesterase
AD: Alzheimer’s disease
CRE: cyclic AMP responsive element
CREB: cAMP response element-binding protein
E2: 17β-estradiol
ER: estrogen receptor
GPR30: G protein-coupled receptor 30
iso-OMPA: tetraisopropylpyro-phosphoramide
miRNA: microRNA
NFs: neurofilaments
NGF: nerve growth factor
PRiMA: proline-rich membrane anchor.
