# Supplementary figures and images for: Genistein, a Phytoestrogen in Soybean, Induces the Expression of Acetylcholinesterase via G Protein-Coupled Receptor 30 in PC12 Cells
Source: Front Mol Neurosci. 2018 Feb 27;11:59. doi: 10.3389/fnmol.2018.00059 (PMC5835133; doi:10.3389/fnmol.2018.00059)

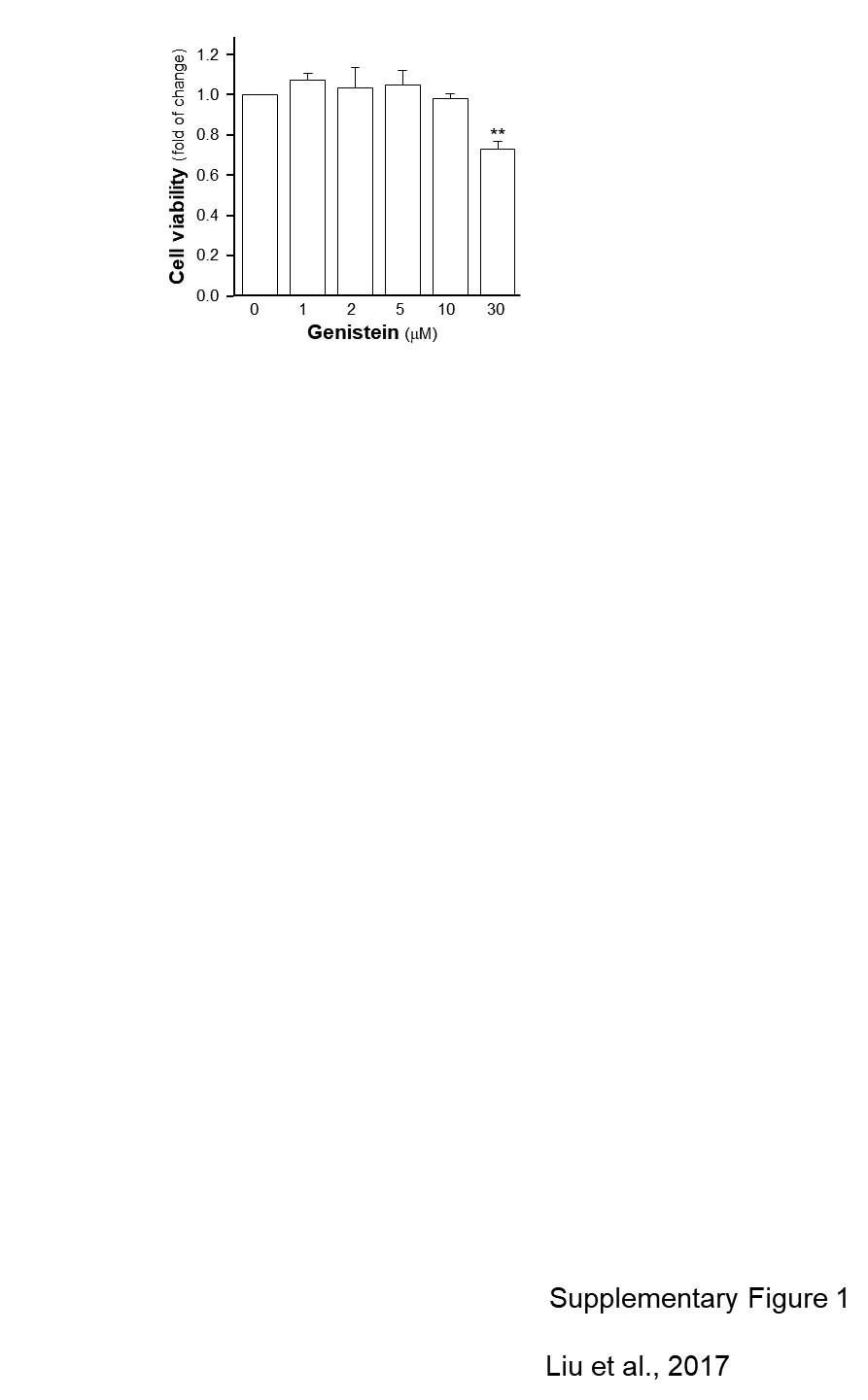


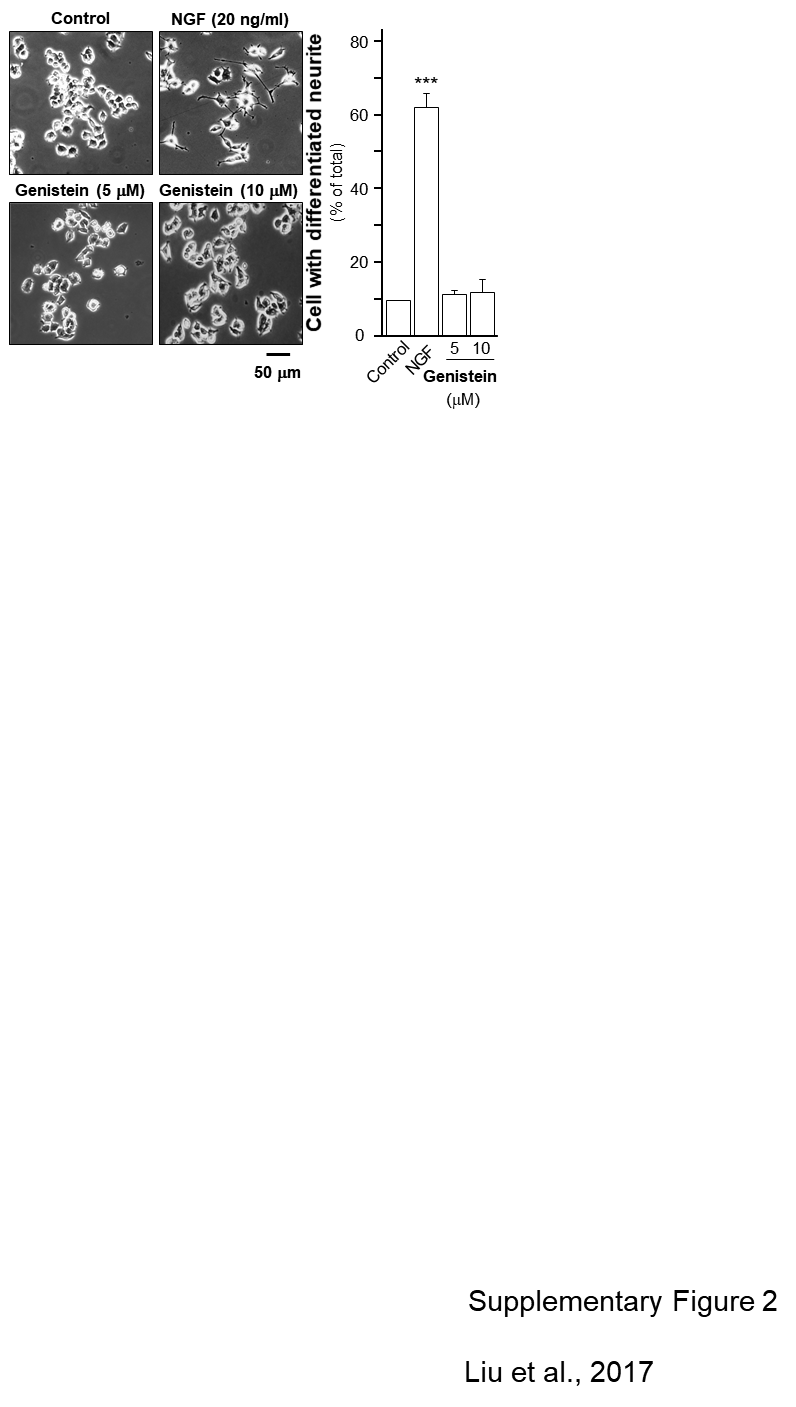

Supplement: FIGURE S1 — Effect of genistein on the viability of PC12 cells. Cultured PC12 cells were treated with the different doses of genistein for 48 h. Cell viability (using the colorimetric MTT assay) was performed. No significant increase in cell viability was observed up to 10 μM. Values are in the fold of change as compared to control, and in mean ± SEM, n = 5, each with triplicate samples. **p < 0.01. [file Data_Sheet_1.docx]
